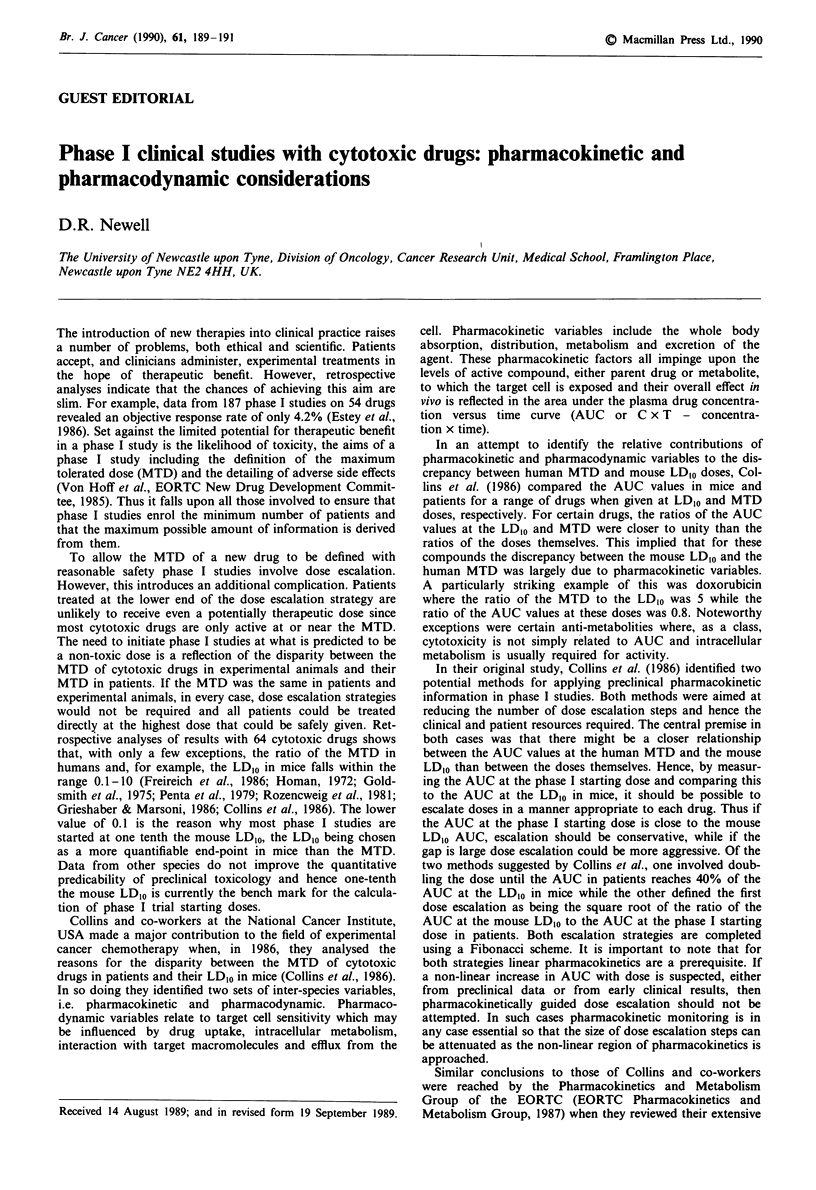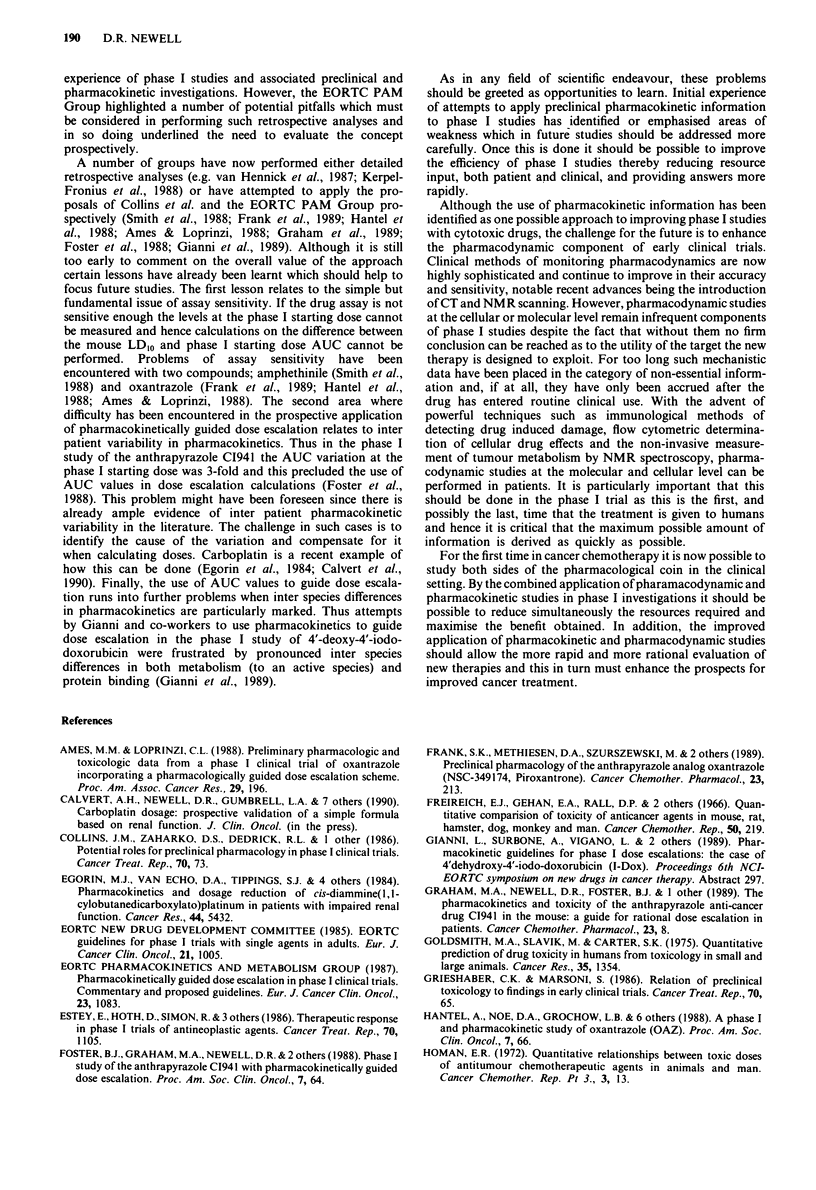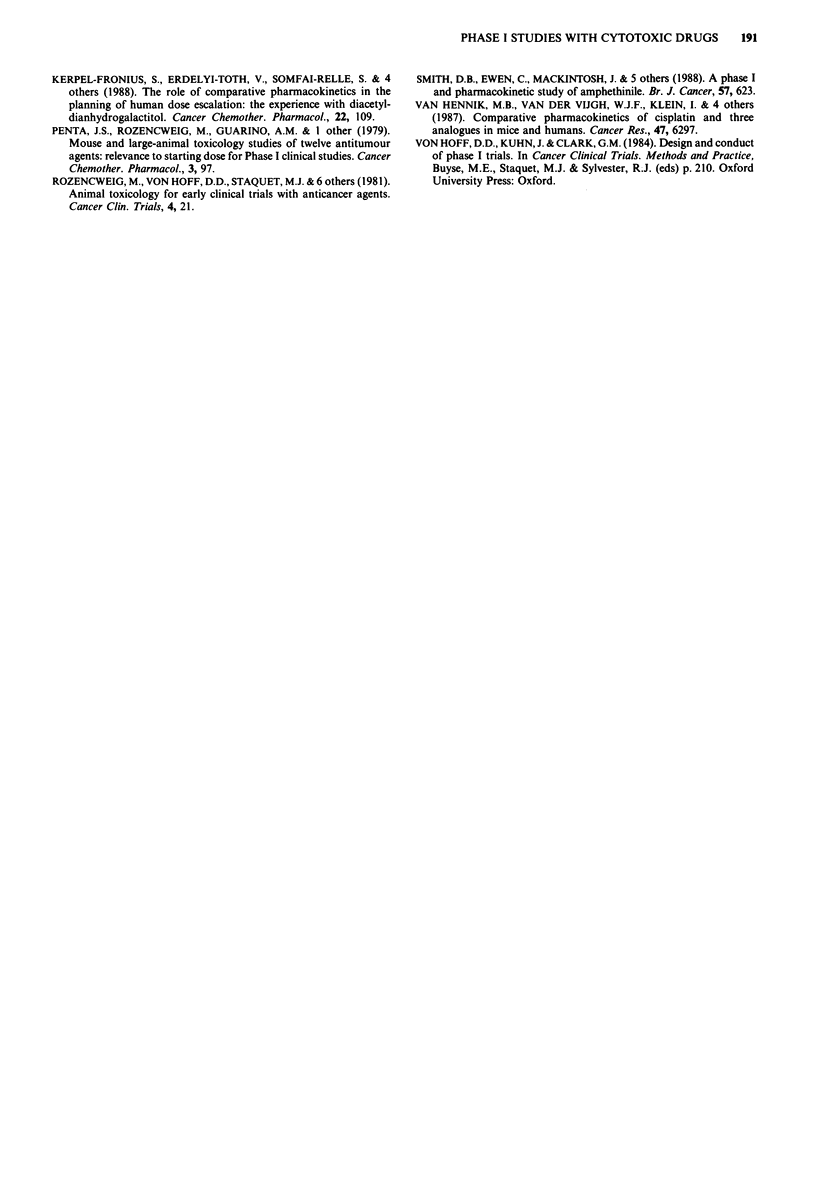# Phase I clinical studies with cytotoxic drugs: pharmacokinetic and pharmacodynamic considerations.

**DOI:** 10.1038/bjc.1990.35

**Published:** 1990-02

**Authors:** D. R. Newell

**Affiliations:** University of Newcastle upon Tyne, Division of Oncology, Medical School, UK.


Br. J. Cancer (1990), 61, 189-191 © Macmillan Press Ltd., 1990

GUEST EDITORIAL

### Phase I clinical studies with cytotoxic drugs: pharmacokinetic and pharmacodynamic considerations

D.R. Newell

*The University of Newcastle upon Tyne, Division of Oncology, Cancer Research Unit, Medical School, Framlington Place, Newcastle upon Tyne NE2 4HH, UK.*

The introduction of new therapies into clinical practice raises a number of problems, both ethical and scientific. Patients accept, and clinicians administer, experimental treatments in the hope of therapeutic benefit. However, retrospective analyses indicate that the chances of achieving this aim are slim. For example, data from 187 phase I studies on 54 drugs revealed an objective response rate of only 4.2% (Estey *et al.*, 1986). Set against the limited potential for therapeutic benefit in a phase I study is the likelihood of toxicity, the aims of a phase I study including the definition of the maximum tolerated dose (MTD) and the detailing of adverse side effects (Von Hoff *et al.*, EORTC New Drug Development Committee, 1985). Thus it falls upon all those involved to ensure that phase I studies enrol the minimum number of patients and that the maximum possible amount of information is derived from them.

To allow the MTD of a new drug to be defined with reasonable safety phase I studies involve dose escalation. However, this introduces an additional complication. Patients treated at the lower end of the dose escalation strategy are unlikely to receive even a potentially therapeutic dose since most cytotoxic drugs are only active at or near the MTD. The need to initiate phase I studies at what is predicted to be a non-toxic dose is a reflection of the disparity between the MTD of cytotoxic drugs in experimental animals and their MTD in patients. If the MTD was the same in patients and experimental animals, in every case, dose escalation strategies would not be required and all patients could be treated directly at the highest dose that could be safely given. Retrospective analyses of results with 64 cytotoxic drugs shows that, with only a few exceptions, the ratio of the MTD in humans and, for example, the $LD_{10}$ in mice falls within the range 0.1-10 (Freireich *et al.*, 1986; Homan, 1972; Goldsmith *et al.*, 1975; Penta *et al.*, 1979; Rozencweig *et al.*, 1981; Grieshaber & Marsoni, 1986; Collins *et al.*, 1986). The lower value of 0.1 is the reason why most phase I studies are started at one tenth the mouse $LD_{10}$, the $LD_{10}$ being chosen as a more quantifiable end-point in mice than the MTD. Data from other species do not improve the quantitative predicability of preclinical toxicology and hence one-tenth the mouse $LD_{10}$ is currently the bench mark for the calculation of phase I trial starting doses.

Collins and co-workers at the National Cancer Institute, USA made a major contribution to the field of experimental cancer chemotherapy when, in 1986, they analysed the reasons for the disparity between the MTD of cytotoxic drugs in patients and their $LD_{10}$ in mice (Collins *et al.*, 1986). In so doing they identified two sets of inter-species variables, i.e. pharmacokinetic and pharmacodynamic. Pharmacodynamic variables relate to target cell sensitivity which may be influenced by drug uptake, intracellular metabolism, interaction with target macromolecules and efflux from the cell. Pharmacokinetic variables include the whole body absorption, distribution, metabolism and excretion of the agent. These pharmacokinetic factors all impinge upon the levels of active compound, either parent drug or metabolite, to which the target cell is exposed and their overall effect *in vivo* is reflected in the area under the plasma drug concentration versus time curve (AUC or $C \times T$ – concentration $\times$ time).

In an attempt to identify the relative contributions of pharmacokinetic and pharmacodynamic variables to the discrepancy between human MTD and mouse $LD_{10}$ doses, Collins *et al.* (1986) compared the AUC values in mice and patients for a range of drugs when given at $LD_{10}$ and MTD doses, respectively. For certain drugs, the ratios of the AUC values at the $LD_{10}$ and MTD were closer to unity than the ratios of the doses themselves. This implied that for these compounds the discrepancy between the mouse $LD_{10}$ and the human MTD was largely due to pharmacokinetic variables. A particularly striking example of this was doxorubicin where the ratio of the MTD to the $LD_{10}$ was 5 while the ratio of the AUC values at these doses was 0.8. Noteworthy exceptions were certain anti-metabolities where, as a class, cytotoxicity is not simply related to AUC and intracellular metabolism is usually required for activity.

In their original study, Collins *et al.* (1986) identified two potential methods for applying preclinical pharmacokinetic information in phase I studies. Both methods were aimed at reducing the number of dose escalation steps and hence the clinical and patient resources required. The central premise in both cases was that there might be a closer relationship between the AUC values at the human MTD and the mouse $LD_{10}$ than between the doses themselves. Hence, by measuring the AUC at the phase I starting dose and comparing this to the AUC at the $LD_{10}$ in mice, it should be possible to escalate doses in a manner appropriate to each drug. Thus if the AUC at the phase I starting dose is close to the mouse $LD_{10}$ AUC, escalation should be conservative, while if the gap is large dose escalation could be more aggressive. Of the two methods suggested by Collins *et al.*, one involved doubling the dose until the AUC in patients reaches 40% of the AUC at the $LD_{10}$ in mice while the other defined the first dose escalation as being the square root of the ratio of the AUC at the mouse $LD_{10}$ to the AUC at the phase I starting dose in patients. Both escalation strategies are completed using a Fibonacci scheme. It is important to note that for both strategies linear pharmacokinetics are a prerequisite. If a non-linear increase in AUC with dose is suspected, either from preclinical data or from early clinical results, then pharmacokinetically guided dose escalation should not be attempted. In such cases pharmacokinetic monitoring is in any case essential so that the size of dose escalation steps can be attenuated as the non-linear region of pharmacokinetics is approached.

Similar conclusions to those of Collins and co-workers were reached by the Pharmacokinetics and Metabolism Group of the EORTC (EORTC Pharmacokinetics and Metabolism Group, 1987) when they reviewed their extensive

Received 14 August 1989; and in revised form 19 September 1989.

experience of phase I studies and associated preclinical and pharmacokinetic investigations. However, the EORTC PAM Group highlighted a number of potential pitfalls which must be considered in performing such retrospective analyses and in so doing underlined the need to evaluate the concept prospectively.

A number of groups have now performed either detailed retrospective analyses (e.g. van Hennick *et al.*, 1987; Kerpel-Fronius *et al.*, 1988) or have attempted to apply the proposals of Collins *et al.* and the EORTC PAM Group prospectively (Smith *et al.*, 1988; Frank *et al.*, 1989; Hantel *et al.*, 1988; Ames & Loprinzi, 1988; Graham *et al.*, 1989; Foster *et al.*, 1988; Gianni *et al.*, 1989). Although it is still too early to comment on the overall value of the approach certain lessons have already been learnt which should help to focus future studies. The first lesson relates to the simple but fundamental issue of assay sensitivity. If the drug assay is not sensitive enough the levels at the phase I starting dose cannot be measured and hence calculations on the difference between the mouse $LD_{10}$ and phase I starting dose AUC cannot be performed. Problems of assay sensitivity have been encountered with two compounds; amphethinile (Smith *et al.*, 1988) and oxantrazole (Frank *et al.*, 1989; Hantel *et al.*, 1988; Ames & Loprinzi, 1988). The second area where difficulty has been encountered in the prospective application of pharmacokinetically guided dose escalation relates to inter patient variability in pharmacokinetics. Thus in the phase I study of the anthrapyrazole CI941 the AUC variation at the phase I starting dose was 3-fold and this precluded the use of AUC values in dose escalation calculations (Foster *et al.*, 1988). This problem might have been foreseen since there is already ample evidence of inter patient pharmacokinetic variability in the literature. The challenge in such cases is to identify the cause of the variation and compensate for it when calculating doses. Carboplatin is a recent example of how this can be done (Egorin *et al.*, 1984; Calvert *et al.*, 1990). Finally, the use of AUC values to guide dose escalation runs into further problems when inter species differences in pharmacokinetics are particularly marked. Thus attempts by Gianni and co-workers to use pharmacokinetics to guide dose escalation in the phase I study of 4'-deoxy-4'-iodo-doxorubicin were frustrated by pronounced inter species differences in both metabolism (to an active species) and protein binding (Gianni *et al.*, 1989).

As in any field of scientific endeavour, these problems should be greeted as opportunities to learn. Initial experience of attempts to apply preclinical pharmacokinetic information to phase I studies has identified or emphasised areas of weakness which in future studies should be addressed more carefully. Once this is done it should be possible to improve the efficiency of phase I studies thereby reducing resource input, both patient and clinical, and providing answers more rapidly.

Although the use of pharmacokinetic information has been identified as one possible approach to improving phase I studies with cytotoxic drugs, the challenge for the future is to enhance the pharmacodynamic component of early clinical trials. Clinical methods of monitoring pharmacodynamics are now highly sophisticated and continue to improve in their accuracy and sensitivity, notable recent advances being the introduction of CT and NMR scanning. However, pharmacodynamic studies at the cellular or molecular level remain infrequent components of phase I studies despite the fact that without them no firm conclusion can be reached as to the utility of the target the new therapy is designed to exploit. For too long such mechanistic data have been placed in the category of non-essential information and, if at all, they have only been accrued after the drug has entered routine clinical use. With the advent of powerful techniques such as immunological methods of detecting drug induced damage, flow cytometric determination of cellular drug effects and the non-invasive measurement of tumour metabolism by NMR spectroscopy, pharmacodynamic studies at the molecular and cellular level can be performed in patients. It is particularly important that this should be done in the phase I trial as this is the first, and possibly the last, time that the treatment is given to humans and hence it is critical that the maximum possible amount of information is derived as quickly as possible.

For the first time in cancer chemotherapy it is now possible to study both sides of the pharmacological coin in the clinical setting. By the combined application of pharamacodynamic and pharmacokinetic studies in phase I investigations it should be possible to reduce simultaneously the resources required and maximise the benefit obtained. In addition, the improved application of pharmacokinetic and pharmacodynamic studies should allow the more rapid and more rational evaluation of new therapies and this in turn must enhance the prospects for improved cancer treatment.

### References

AMES, M.M. & LOPRINZI, C.L. (1988). Preliminary pharmacologic and toxicologic data from a phase I clinical trial of oxantrazole incorporating a pharmacologically guided dose escalation scheme. *Proc. Am. Assoc. Cancer Res.*, **29**, 196.

CALVERT, A.H., NEWELL, D.R., GUMBRELL, L.A. & 7 others (1990). Carboplatin dosage: prospective validation of a simple formula based on renal function. *J. Clin. Oncol.* (in the press).

COLLINS, J.M., ZAHARKO, D.S., DEDRICK, R.L. & 1 other (1986). Potential roles for preclinical pharmacology in phase I clinical trials. *Cancer Treat. Rep.*, **70**, 73.

EGORIN, M.J., VAN ECHO, D.A., TIPPINGS, S.J. & 4 others (1984). Pharmacokinetics and dosage reduction of *cis*-diammine(1,1-cylobutanedicarboxylato)platinum in patients with impaired renal function. *Cancer Res.*, **44**, 5432.

EORTC NEW DRUG DEVELOPMENT COMMITTEE (1985). EORTC guidelines for phase I trials with single agents in adults. *Eur. J. Cancer Clin. Oncol.*, **21**, 1005.

EORTC PHARMACOKINETICS AND METABOLISM GROUP (1987). Pharmacokinetically guided dose escalation in phase I clinical trials. Commentary and proposed guidelines. *Eur. J. Cancer Clin. Oncol.*, **23**, 1083.

ESTEY, E., HOTH, D., SIMON, R. & 3 others (1986). Therapeutic response in phase I trials of antineoplastic agents. *Cancer Treat. Rep.*, **70**, 1105.

FOSTER, B.J., GRAHAM, M.A., NEWELL, D.R. & 2 others (1988). Phase I study of the anthrapyrazole CI941 with pharmacokinetically guided dose escalation. *Proc. Am. Soc. Clin. Oncol.*, **7**, 64.

FRANK, S.K., METHIESEN, D.A., SZURSZEWSKI, M. & 2 others (1989). Preclinical pharmacology of the anthrapyrazole analog oxantrazole (NSC-349174, Piroxantrone). *Cancer Chemother. Pharmacol.*, **23**, 213.

FREIREICH, E.J., GEHAN, E.A., RALL, D.P. & 2 others (1966). Quantitative comparision of toxicity of anticancer agents in mouse, rat, hamster, dog, monkey and man. *Cancer Chemother. Rep.*, **50**, 219.

GIANNI, L., SURBONE, A., VIGANO, L. & 2 others (1989). Pharmacokinetic guidelines for phase I dose escalations: the case of 4'dehydroxy-4'-iodo-doxorubicin (I-Dox). *Proceedings 6th NCI-EORTC symposium on new drugs in cancer therapy*. Abstract 297.

GRAHAM, M.A., NEWELL, D.R., FOSTER, B.J. & 1 other (1989). The pharmacokinetics and toxicity of the anthrapyrazole anti-cancer drug CI941 in the mouse: a guide for rational dose escalation in patients. *Cancer Chemother. Pharmacol.*, **23**, 8.

GOLDSMITH, M.A., SLAVIK, M. & CARTER, S.K. (1975). Quantitative prediction of drug toxicity in humans from toxicology in small and large animals. *Cancer Res.*, **35**, 1354.

GRIESHABER, C.K. & MARSONI, S. (1986). Relation of preclinical toxicology to findings in early clinical trials. *Cancer Treat. Rep.*, **70**, 65.

HANTEL, A., NOE, D.A., GROCHOW, L.B. & 6 others (1988). A phase I and pharmacokinetic study of oxantrazole (OAZ). *Proc. Am. Soc. Clin. Oncol.*, **7**, 66.

HOMAN, E.R. (1972). Quantitative relationships between toxic doses of antitumour chemotherapeutic agents in animals and man. *Cancer Chemother. Rep. Pt 3.*, **3**, 13.

KERPEL-FRONIUS, S., ERDELYI-TOTH, V., SOMFAI-RELLE, S. & 4 others (1988). The role of comparative pharmacokinetics in the planning of human dose escalation: the experience with diacetyl-dianhydrogalactitol. *Cancer Chemother. Pharmacol.*, **22**, 109.

PENTA, J.S., ROZENCWEIG, M., GUARINO, A.M. & 1 other (1979). Mouse and large-animal toxicology studies of twelve antitumour agents: relevance to starting dose for Phase I clinical studies. *Cancer Chemother. Pharmacol.*, **3**, 97.

ROZENCWEIG, M., VON HOFF, D.D., STAQUET, M.J. & 6 others (1981). Animal toxicology for early clinical trials with anticancer agents. *Cancer Clin. Trials*, **4**, 21.

SMITH, D.B., EWEN, C., MACKINTOSH, J. & 5 others (1988). A phase I and pharmacokinetic study of amphethinile. *Br. J. Cancer*, **57**, 623.

VAN HENNIK, M.B., VAN DER VIJGH, W.J.F., KLEIN, I. & 4 others (1987). Comparative pharmacokinetics of cisplatin and three analogues in mice and humans. *Cancer Res.*, **47**, 6297.

VON HOFF, D.D., KUHN, J. & CLARK, G.M. (1984). Design and conduct of phase I trials. In *Cancer Clinical Trials. Methods and Practice*, Buyse, M.E., Staquet, M.J. & Sylvester, R.J. (eds) p. 210. Oxford University Press: Oxford.